# Occurrence of *Schistosoma bovis* on Pemba Island, Zanzibar: implications for urogenital schistosomiasis transmission monitoring

**DOI:** 10.1017/S0031182018001154

**Published:** 2018-08-08

**Authors:** Tom Pennance, Shaali M. Ame, Armour Khamis Amour, Khamis Rashid Suleiman, Fiona Allan, David Rollinson, Bonnie L. Webster

**Affiliations:** 1 Natural History Museum, Cromwell Road, London SW75BD, UK; 2 London Centre for Neglected Tropical Disease Research, Imperial College London, School of Public Health, Norfolk Pl, Paddington, London W2 1PG, UK; 3 Cardiff University, Cardiff CF10 3AT, UK; 4 Public Health Laboratory, Chake Chake, Pemba, United Republic of Tanzania

**Keywords:** *Bulinus*, cattle, Pemba, *Schistosoma bovis*, *Schistosoma haematobium*, schistosomiasis, schistosomes, snails, Zanzibar

## Abstract

The causative agent of urogenital schistosomiasis, *Schistosoma haematobium*, was thought to be the only schistosome species transmitted through *Bulinus* snails on Unguja and Pemba Island (Zanzibar, United Republic of Tanzania). For insights into the environmental risk of *S. haematobium* transmission on Pemba Island, malacological surveys collecting *Bulinus globosus* and *B. nasutus*, two closely related potential intermediate hosts of *S. haematobium* were conducted across the island in November 2016. Of 1317 *B. globosus*/*B. nasutus* collected, seven *B. globosus*, identified through sequencing a DNA region of the mitochondrial cytochrome oxidase subunit 1 (*cox*1), were observed with patent infections assumed to be *S. haematobium*. However, when the collected cercariae were identified through sequencing a region of the *cox*1 and the nuclear internal transcribed spacer (ITS1 + 2), schistosomes from five of these *B. globosus* collected from a single locality were in fact *S. bovis*. The identified presence of *S. bovis* raises concerns for animal health on Pemba, and complicates future transmission monitoring of *S. haematobium*. These results show the pertinence for not only sensitive, but also species-specific markers to be used when identifying cercariae during transmission monitoring, and also provide the first molecular confirmation for *B. globosus* transmitting *S. bovis* in East Africa.

## Introduction

The snail-borne neglected tropical disease (NTD), schistosomiasis, is the most important freshwater parasitic disease of humans associated with poverty, poor sanitation and lack of safe water supplies (Steinmann *et al*., 2006; Hotez *et al*., 2014), with an estimated 180–200 million people primarily from low- and middle-income countries being infected (GBD 2016 Disease and Injury Incidence and Prevalence Collaborators, 2017). Ambitious goals to eliminate schistosomiasis have been announced by the WHO as part of its roadmap to overcome the global impact of NTDs by 2020–2025 (WHO, 2012). Whilst mass drug administration, behavioural change through education and snail control are having a major impact on schistosomiasis, further research into schistosome transmission biology together with better tools for transmission monitoring and surveillance are required to help achieve and monitor the success of these ambitious goals (Stothard *et al*., 2017). Schistosomiasis is also a disease of animals, with large numbers of domestic livestock affected worldwide but the actual veterinary and economic impact is largely unknown (De Bont and Vercruysse, 1997, 1998).

There are 25 recognized species of mammalian schistosomes that cause human and animal infections, which can be split into four *Schistosoma* species groups (Webster *et al.*, 2006). The largest group is the *Schistosoma haematobium* group containing nine species that are all transmitted through *Bulinus* snails (Brown, 1994) with two species, *S. haematobium* and *S. bovis*, being responsible for the majority of all human (Hotez and Kamath, 2009) and livestock infections (De Bont and Vercruysse, 1997), respectively. Central to this group is *S. haematobium*, a major human schistosome species being the most widespread and prevalent across Africa and solely responsible for human urogenital schistosomiasis with often severe pathology (Schwartz, 1981; Leutscher *et al*., 2000; Bustinduy *et al*., 2014; Kjetland *et al*., 2014; Christinet *et al*., 2016). *Schistosoma bovis* is a pathogen of domestic livestock and some artiodactylids (Standley *et al*., 2012), with its distribution commonly overlapping with that of *S. haematobium* across mainland Africa (Moné *et al*., 1999), and utilising a wide range of *Bulinus* (Southgate and Knowles, 1975*a*, 1975*b*; Stothard *et al*., 2004). These two species, among others, are also able to hybridize and inter-specific hybridization is now recognized in West Africa with possible detrimental consequences on disease control (Huyse *et al*., 2009; Webster *et al*., 2013; Léger and Webster, 2017).

Pemba and Unguja Islands (Zanzibar Archipelago, United Republic of Tanzania) have been historically identified as ‘model islands’ for implementing multiple effective infectious disease control and elimination programmes in sub-Saharan Africa (Pennance *et al*., 2016). For schistosomiasis control, Zanzibar also offers an advantage due to the allopatric transmission of *S. haematobium* through a single snail host, *Bulinus globosus*, on both Islands (Stothard *et al*., 2000), whereas across most of sub-Saharan Africa, multiple *Schistosoma* and *Bulinus* species occur in sympatry (Brown, 1994), complicating control interventions and surveillance. Urogenital schistosomiasis was highly endemic on both islands but is now targeted for elimination (Knopp *et al*., 2012, 2013).

As we move towards or reach elimination, there becomes a need for more sensitive methods to monitor the levels of transmission when egg–patent human infections become scarce (Le and Hsieh, 2017; Stothard *et al*., 2017), the risk of infection and also a way to prove transmission interruption when it is finally reached. Xenomonitoring is a nucleic acid-based molecular diagnostic used to monitor the transmission of several vector-borne diseases (Cunningham *et al*., 2016; Minetti *et al*., 2016; Cook *et al*., 2017), including to some extent schistosomiasis where tools are being developed for the xenomonitoring of snails that could support schistosomiasis transmission and elimination monitoring (Hamburger *et al*., 2004; Allan *et al*., 2013; Lu *et al*., 2016; Abbasi *et al*., 2017). The first stage for snail xenomonitoring for schistosomiasis is the identification of patent schistosome infections within the snails and collecting cercariae shed from them. Here, we report on the molecular identification of these cercariae and the infected snails collected from Pemba Island (Zanzibar) and how the findings complicate the development of robust molecular xenomonitoring protocols for ongoing and future transmission monitoring.

## Methods

### Malacological surveys and *Schistosoma* collection

In November 2016, as part of a larger ongoing molecular xenomonitoring study on Pemba, *Bulinus* snails were collected, by scooping, from human freshwater contact sites in eight shehias (smallest division of administrative regions), examined and individually induced to shed cercariae following previous methods (Allan *et al*., 2013). An experienced microscopist identified schistosome cercariae, which were individually pipetted in 3.5 *µ*L aliquots onto Whatman FTA cards (Whatman, Part of GE Healthcare, Florham Park, USA) for long-term deoxyribonucleic acid (DNA) storage. After shedding, all infected snails were preserved in 100% ethanol for future morphological and molecular characterization.

### *Schistosoma* and *Bulinus* identification

DNA from individual cercariae was eluted from the FTA cards (Webster *et al*., 2015) and characterized by amplification and sequencing of the mitochondrial cytochrome oxidase subunit 1 (*cox*1) and partial nuclear internal transcribed spacer (ITS1 + 2) DNA regions (Webster *et al*., 2012).

To determine the species of the infected snails, total genomic DNA was extracted from the whole snail tissue using the DNeasy Blood & Tissue Kit (Qiagen, Manchester, UK), with minor changes to the standard protocol in that quantities of the digest reagents were doubled and digests were incubated for at least 12 h. From each snail, a 623 base pair region of the mitochondrial *cox*1 gene was amplified and Sanger sequenced using primers BulCox1 and CO2 following previous protocols (Kane *et al*., 2008). The sequence data were manually edited in Sequencher v5.1 (http://genecodes.com) before being compared with reference sequence databases for *Bulinus* (Kane *et al*., 2008) and *Schistosoma* (Webster *et al*., 2012, 2013) to confirm species.

## Results

**Fig. 1:**
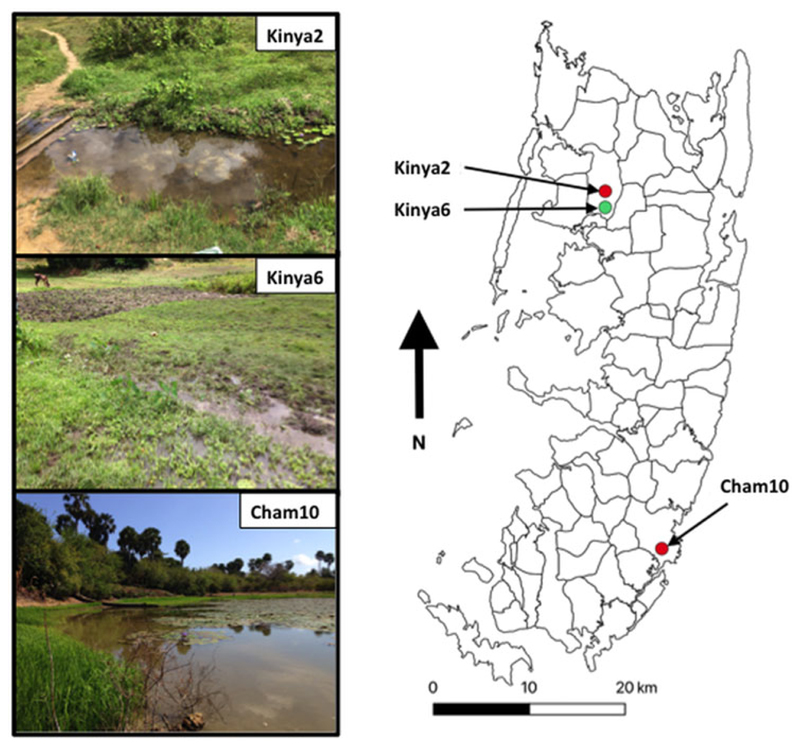
Map outlining shehias (smallest division of administrative regions) on Pemba Island, Zanzibar (United Republic of Tanzania) showing the location and images of two freshwater bodies in Kinyasini (Kinya2 and Kinya6) and one in Chambani (Cham10) where *Schistosoma haematobium* (red) and *Schistosoma bovis* (green) cercariae were recovered from *Bulinus globosus*. GPS coordinates for sites (latitude and longitude in decimal degrees): Kinya2 (−5.02033°, 39.73855°); Kinya6 (−5.03560°, 39.73850°); Cham10 (−5.35805°, 39.79182°).

In total, 1317 *B. globosus* and *B. nasutus* were collected, seven of these snails (Table 1) from Kinyasini (6) and Chambani (1) shehia were shedding schistosome cercariae (Fig. 1). The infected snails were identified as *B. globosus* with two *cox*1 haplotypes recognized (GenBank accession numbers: MH014040 and MH014041) which matched those snails previously reported from Pemba (Kane *et al*., 2008). Cercariae collected from these were assumed initially to be the human parasite *S. haematobium*; however, molecular characterizations of the cercariae from five of these snails, collected from a stream in Kinyasini (Kinya6), were identified as *S. bovis* (Table 1). Two different *S. bovis cox*1 haplotypes [Genbank accessions: *S.b* (i) MH014042 and *S.b* (ii) MH014043] (Table 1) were identified from these five snails; three snails producing *S. bovis* cercariae of a single haplotype and two snails producing *S. bovis* cercariae of both haplotypes suggesting that they had been infected by more than one miracidium.

The other two infected snails shed *S. haematobium* cercariae and were collected from a pond in Chambani (Cham10) and a different stream site in Kinyasini (Kinya2). The *S. haematobium* cercariae from Kinyasini and Chambani, respectively, were of two different *S. haematobium cox*1 haplotypes [Genbank accessions: *S.h* (i) MH014046 and *S.h* (ii) MH014045] with only single haplotypes produced from each snail. These haplotypes matched those identified as group 2 *S. haematobium cox*1 haplotypes found only in the Indian Ocean Islands (Webster *et al*., 2012).

ITS1 + 2 profiles showed no intra-species variation (Genbank Accessions: *S.h* MH014047 and *S.b* MH014044) and were identified as either *S. bovis* or *S. haematobium* by the three inter-specific single nucleotide polymorphisms (Webster *et al*., 2012).

## Discussion

The detection of *S. bovis* on Pemba Island poses a potentially new threat to domestic livestock and wildlife health in Zanzibar (De Bont and Vercruysse, 1997, 1998; Standley *et al*., 2012). The site where *S. bovis* transmission was identified had grazing cattle (see Fig. 1, Kinya6) in close proximity to the water where the shedding snails were collected; therefore, it is quite likely that ongoing transmission is being maintained. Moreover, the movement of infected cattle could enable the spread of the infection particularly as *B. globosus* are found throughout most of the island (Stothard *et al*., 1997).

The presence of *S. bovis* complicates the monitoring of *S. haematobium* transmission since both parasites are shown here to infect the same intermediate snail host and cannot be distinguished from each other easily by microscopy. Therefore, *S. bovis*-infected *B. globosus* could be falsely identified as infected with *S. haematobium*, or *vice-versa*, complicating urogenital schistosomiasis transmission monitoring. This accentuates the need for routine molecular identification of schistosome infections in snails during malacological surveys (Minetti *et al*., 2016), and the development of more species-specific xenomonitoring tools to differentiate *S. bovis* and *S. haematobium* transmission (Webster *et al*., 2010; Abbasi *et al*., 2017). The identification of schistosome cercariae shed from snails is often presumed to be of a particular species due to the snail host involved or the locality of the transmission. Our findings strongly emphasize that these assumptions are not accurate and transmission dynamics of different species may change over time and space. The assumed transmission of only *S. haematobium* by *B. globosus* on Zanzibar and the non-identification of these *S. bovis* infections would have led us to believe that the level of *S. haematobium* transmission is much higher than it actually is, hampering ongoing and future urogenital schistosomiasis transmission monitoring and surveillance.

*Schistosoma haematobium* and *S. bovis* hybridization has also been detected in sympatric West African areas (Webster *et al*., 2013). Zanzibar was considered to be an allopatric area for *S. haematobium* (Webster *et al*., 2012) but the identification of this sympatry with *S. bovis* could, in time, lead to inter-species hybridization. The potential consequences of hybridization include increased host associations of hybrids, possible zoonotic transmission and hybrid vigour (Huyse *et al*., 2009; Webster *et al*., 2013; Léger and Webster, 2017). Investigating the origin of *S. bovis* being transmitted on Pemba, by genetic comparison with other mainland strains of *S. bovis*, may help elucidate how this parasite has been imported to Zanzibar. Since the eradication of the tsetse fly, the vector of human and African animal trypanosomiasis, on Unguja Island (Vreysen *et al*., 2000), there has been an increase of cattle farming (Mdoe, 2003) facilitated by the import of cattle under strict guidelines of the United Republic of Tanzania’s Animal Resources Management Act (1999). Bovine schistosomiasis however is widely ignored/unknown as a veterinary health problem, and therefore is currently not included in these guidelines. This oversight could offer some explanation to how and within what time scale the introduction, or multiple introductions, of *S. bovis* may have occurred. Additionally, the prevalence and intensity of *S. bovis* in local cattle and other potential artiodactylid hosts (Standley *et al*., 2012), such as the Ader’s duiker (*Cephalophus adersi*) endemic to Zanzibar, should be determined to assess the impact on livestock and wildlife health. However, diagnosing *S. bovis* from the definitive host remains challenging, with the detection of *S. bovis* eggs in the stool being difficult and the more sensitive method of observing adult worms in the host being only possible post-mortem *via* dissection. An antigen-based test with promising diagnostic performance has been developed (de la Torre-Escudero *et al*., 2012), which could offer a sensitive method for judging the epidemiology of *S. bovis* in Pemba.

Due to the difficulty in classifying species within the *Bulinus africanus* species complex (Kane *et al*., 2008), previous findings on snail–schistosome compatibilities should be treated with some caution. This molecular confirmation of *B. globosus* naturally transmitting *S. bovis* in East Africa gives credibility to a previous observation (Mwambungu, 1988), and dispels previous claims of *B. globosus* being naturally refractory (Christensen *et al*., 1983) or only an intermediate host in West Africa (Diaw and Vassiliades, 1987; Ndifon *et al*., 1988). Previous evidence for compatibility of *B. nasutus* with *S. bovis* in East Africa is also tainted with contradicting evidence, some showing natural infections (Dowdeswell, 1938; Kinoti, 1964*b*) going against failed experimental infections (Southgate and Knowles, 1975*a*, 1975*b*; Southgate *et al*., 1980) and a lack of naturally infected *B. nasutus* in other endemic areas (Kinoti, 1964*a*; Southgate *et al*., 1980; Mutani *et al*., 1983). It is likely that *S. bovis* has a broad intermediate host range in East Africa utilising several *Bulinus* species, as it has also been identified from *B. ugandae* (Malek, 1969), *B. africanus* (McClelland, 1955; Teesdale and Nelson, 1958; Kassuku *et al*., 1986) and *B. forskalii* (McClelland, 1955). Therefore, studies to confirm the intermediate snail host vectoral capacity and specificity of *S. bovis* to *B. globosus* or indeed other endemic *Bulinus* species on Pemba, including *B. nasutus* and *B. forskalii*, are required to determine the transmission potential and possible spread of this emerging schistosome in Zanzibar.

## Figures and Tables

**Table 1:** Showing the collection sites and genetic profiles of the *Bulinus* and schistosome cercariae analysed

				*Schistosoma* cercariae mitochondrial and nuclear genetic profile	
*Bulinus globosus* ID (*cox*1 haplotype)	Shehia	Site (water body type)	*Schistosoma* cercariae species	*cox*1	ITS1 + 2
Kin2.1 (a)	Kinyasini	Kinya2 (stream)	*S. haematobium*	*S.h* (*i*)	*S.h*

Kin6.1 (b)	Kinyasini	Kinya6 (stream)	*S. bovis*	*S.b* (*i*)	*S.b*

Kin6.2 (b)	Kinyasini	Kinya6 (stream)	*S. bovis*	*S.b* (*i* & *ii*)	*S.b*

Kin6.3 (b)	Kinyasini	Kinya6 (stream)	*S. bovis*	*S.b* (*i* & *ii*)	*S.b*

Kin6.4 (b)	Kinyasini	Kinya6 (stream)	*S. bovis*	*S.b* (*i*)	*S.b*

Kin6.5 (b)	Kinyasini	Kinya6 (stream)	*S. bovis*	*S.b* (*ii*)	*S.b*

Cham10.1 (b)	Chambani	Cham10 (pond)	*S. haematobium*	*S.h* (*ii*)	*S.h*

Two *Bulinus globosus cox*1 haplotypes [Genbank accessions: (a) MH014040 and (b) MH014041]. Two *S. haematobium cercariae cox*1 haplotypes, Genbank accessions: *S.h* (i) MH014046 and *S.h* (ii) MH01404 and the two *S. bovis cox*1 haplotypes, Genbank accessions: *S.b* (i) MH014042 and *S.b* (ii) MH014043. ITS1 + 2 profiles showed no intra species variation (Genbank accessions: *S.h* MH014047 and *S.b* MH014044).
